# Increased both cortical activation and functional connectivity after transcranial direct current stimulation in patients with post-stroke: A functional near-infrared spectroscopy study

**DOI:** 10.3389/fpsyt.2022.1046849

**Published:** 2022-12-07

**Authors:** Caihong Yang, Tingyu Zhang, Kaiqi Huang, Menghui Xiong, Huiyu Liu, Pu Wang, Yan Zhang

**Affiliations:** ^1^Department of Rehabilitation Medicine, The Seventh Affiliated Hospital, Sun Yat-sen University, Shenzhen, Guangdong, China; ^2^School of Psychology, Central China Normal University, Wuhan, Hubei, China; ^3^The Seventh Affiliated Hospital, Sun Yat-sen University, Guangzhou, Guangdong, China; ^4^Brain Cognition and Brain Disease Institute (BCBDI), Shenzhen Institutes of Advanced Technology, Chinese Academy of Sciences, Shenzhen, China; ^5^Department of Rehabilitation Medicine, Yue Bei People’s Hospital, Shaoguan, Guangdong, China; ^6^Department of Rehabilitation Medicine, Tianyang District People’s Hospital, Baise, Guangxi, China; ^7^School of Educational Science, Huazhong University of Science and Technology, Wuhan, Hubei, China

**Keywords:** post-stroke patients, transcranial direct current stimulation (tDCS), functional near-infrared spectroscopy (fNIRS), cognitive impairment (CI), cortical activation, functional connectivity

## Abstract

**Background:**

Previous studies have shown that cognitive impairment is common after stroke. Transcranial direct current stimulation (tDCS) is a promising tool for rehabilitating cognitive impairment. This study aimed to investigate the effects of tDCS on the rehabilitation of cognitive impairment in patients with stroke.

**Methods:**

Twenty-two mild–moderate post-stroke patients with cognitive impairments were treated with 14 tDCS sessions. A total of 14 healthy individuals were included in the control group. Cognitive function was assessed using the Mini-Mental State Examination (MMSE) and the Montreal Cognitive Assessment (MoCA). Cortical activation was assessed using functional near-infrared spectroscopy (fNIRS) during the verbal fluency task (VFT).

**Results:**

The cognitive function of patients with stroke, as assessed by the MMSE and MoCA scores, was lower than that of healthy individuals but improved after tDCS. The cortical activation of patients with stroke was lower than that of healthy individuals in the left superior temporal cortex (lSTC), right superior temporal cortex (rSTC), right dorsolateral prefrontal cortex (rDLPFC), right ventrolateral prefrontal cortex (rVLPFC), and left ventrolateral prefrontal cortex (lVLPFC) cortical regions. Cortical activation increased in the lSTC cortex after tDCS. The functional connectivity (FC) between the cerebral hemispheres of patients with stroke was lower than that of healthy individuals but increased after tDCS.

**Conclusion:**

The cognitive and brain functions of patients with mild-to-moderate stroke were damaged but recovered to a degree after tDCS. Increased cortical activation and increased FC between the bilateral cerebral hemispheres measured by fNIRS are promising biomarkers to assess the effectiveness of tDCS in stroke.

## Introduction

Post-stroke cognitive impairment is a common complication after a stroke and an important risk predictor of declining quality of life (1, 2). For example, up to 55% of patients with stroke have episodic memory deficits, 40% have executive function deficits, and 23% have language deficits (3). The high prevalence of cognitive dysfunction after stroke makes cognitive rehabilitation interventions crucial.

After a stroke, people have been found to have abnormal cortical activation in the brain, which is associated with persistent functional impairment. Several studies have found that the extent of cortical activation in patients with stroke is related to the degree of functional damage (4, 5). In addition, the balance between the cerebral hemispheres of patients with stroke is destroyed; the decreased excitability of one hemisphere cortex may lead to the enhancement of the opposite hemisphere’s cortex (6). The competition model between cerebral hemispheres explains this phenomenon, showing that homologous regions in healthy brains inhibit each other. When a stroke occurs, this interhemispheric inhibition (IHI) is destroyed, leading to disinhibition of the contralateral cortex and excessive activation, further reducing brain function (7–9). Thus, according to the competition model between cerebral hemispheres, the functional connectivity (FC) between the bilateral cerebral hemispheres is weakened or increased when a stroke occurs. Caliandro analyzed the small-world properties of the resting-state FC of patients with acute stroke using electroencephalography (EEG) and found that the increase or decrease of the small-world properties in patients with stroke depends on the frequency band analyzed (10). Lu and Arun used task-state FC based on functional near-infrared spectroscopy (fNIRS). They found that compared with the resting state, the FC of the stroke group increased during the task state (11). Compared with the healthy group, the FC of the stroke group during the task state was increased (11, 12). Hence, there is no consensus regarding this issue. In addition, it is critical to regulating stroke based on the levels of cortical activation due to abnormal brain cortical activation. Abnormal FC is very common in stroke patients with cognitive impairment.

Transcranial direct current stimulation (tDCS) is a non-invasive brain stimulation technique (13) that has been widely used to improve cognitive function in patients with stroke (2, 14). Previous studies indicated three different electrode placement schemes for tDCS. First, the anode electrode was placed above the affected cerebral hemisphere to increase the excitability of neurons; the cathode was set as the reference electrode. Second, the cathode was placed above the healthy hemisphere to reduce the excitability of neurons; the anode was set as the reference electrode. Third, the anode was placed on the affected side and the cathode on the healthy side to balance the excitability of neurons in both hemispheres (15–17). In this study, we placed the anodes and cathodes on F3 and F4 electrodes (10–20 EEG electrodes) with a 2 mA intensity.

Some research has shown that using tDCS with anodes and cathodes on F3 and F4 electrodes can improve cognitive function in patients with stroke. Baker found that anodal tDCS with anodes and cathodes on F3 and F4 electrodes enhanced naming accuracy in patients with stroke (18). Park found that using anode tDCS and cognitive rehabilitation training simultaneously positively impacted mild-to-moderate cognitive dysfunction in patients with stroke (19). Shaker used tDCS/sham tDCS to investigate the effect of tDCS on cognitive functions in patients with stroke. They found that cognitive functions were higher after tDCS than sham tDCS in terms of attention and concentration, figural memory, logical reasoning, and reaction behavior (2).

Using tDCS with the anodes and cathodes on F3 and F4 electrodes can improve the cognitive ability of individuals with cognitive impairment after stroke (20) and change their cortical excitability (21). In animal studies (22, 23), direct currents have been shown to change brain excitability. Based on this discovery in animal models, Nitsche and Paulus conducted a study on tDCS in humans (23). They found that anodic stimulation increased cortical excitability, whereas cathodic stimulation decreased it. Due to IHI after stroke, tDCS can increase ipsilateral and reduce contralateral excitability (24). For example, Feltman and Sarkis found that placing anodes and cathodes on F3 and F4 electrodes with 2 mA intensity could activate the dorsolateral prefrontal cortex (DLPFC) and enhance cognitive function (25–27). Some studies have reported that tDCS positively affects the treatment of patients with stroke (24, 28). This study aimed to investigate whether the cortical activation of patients with stroke after anodic tDCS would change. We hypothesized that anodic tDCS would increase cortical excitability in the left cerebral hemisphere.

Using tDCS with anodes and cathodes on F3 and F4 electrodes also changed FC in patients with stroke. Some studies using fMRI technology have found that tDCS can widely regulate interhemispheric connectivity in patients with stroke (29), increasing connectivity between the hemispheres (30, 31). This study aimed to investigate whether the FC of patients with stroke after anodic tDCS would be changed. We hypothesized that anodic tDCS would increase the FC between the bilateral cerebral hemispheres.

Various techniques can be used to observe the cortical activation in the brain. Several characteristics of fNIRS, a non-invasive optical technique, including portability, non-invasive, cost-effectiveness, and tolerance, make it a favorable tool for clinical nursing and neuroscience research (32). fNIRS can indirectly detect the oxy-hemoglobin (oxy-Hb) and the deoxy-hemoglobin (deoxy-Hb) concentration change in particular brain regions of the participants while performing tasks. fNIRS observes the activation of the cerebral cortex by determining the intensity of the scattered light in the cerebral cortex (33). The cortical activity observed based on fNIRS can also be used to further study brain functional connectivity (34, 35). Regarding the identification of oxy-Hb vs. deoxy-Hb indicators, oxy-Hb has a stronger signal amplitude than deoxy-Hb, thus, the former is often used for fNIRS research. Several neuroimaging studies have consistently shown that mild cognitive impairment and dementia involve reduced cerebral blood flow compared to normal cognition controls. This is largely localized to the medial temporal lobe, posterior cingulate, prefrontal cortex (PFC), and inferior parietal cortex (36–38). It is generally believed that oxy-Hb changes are important indicators of activity intensity and cognitive function in the brain. fNIRS has also been used to study cortical activation in patients with stroke. For example, Kim used fNIRS to study the oxy-Hb changes in patients with stroke in robotic mirror therapy to observe the efficacy of the therapy (39). Mihara also used fNIRS to study cortical activation differences between patients with stroke and healthy peers during ataxia gait (40). Therefore, this study used fNIRS to study the oxy-Hb changes in cortical activation and FC after tDCS in patients with stroke.

The verbal fluency task (VFT) has been used to measure executive functions (41). There are indications that the sensitivity of fNIRS is sufficient to detect small metabolic changes during the execution of cognitive tasks, including the VFT of letters or categories (42). Moreover, the VFT is the most widely used task with impaired understanding activation (43). When participants performed the VFT, the prefrontal cortex was extensively activated, especially the DLPFC (2, 44). A series of studies have found that lesions after post-stroke may be widespread in the frontal and temporal regions (45, 46). Therefore, this study used the VFT to measure executive function in stroke patients with cognitive impairment.

## Materials and methods

### Participants

There were 22 patients with mild–moderate stroke (15 men and 7 women) and 14 healthy individuals (10 men and 4 women). Twenty-two mild–moderate stroke patients with post-stroke cognitive impairment met the criteria of stroke diagnosis and treatment guidelines. The onset time was within 2 weeks to 6 months. Education level was higher than 6 years. The cognitive impairment occurred after the stroke. Their National Institutes of Health Stroke Scale (NIHSS) score on admission was less than 20 (47). Those with severe visual impairment, hearing impairment, and other factors affecting cognitive examination such as sensory aphasia were excluded. All patients were right-handed. The demographic data of the participant are shown in Table 1.

**TABLE 1:** Demographic data of the participant.

Characteristic	ST	HI	*t*	*df*	*p*
*N*	22	14			NA
Age (mean ± SD)	60.91 ± 8.79	55.5 ± 4.958	1.875	34	0.070
Sex (mean ± SD)			0.623	34	0.538
MMSE (mean ± SD)	20 ± 4.739	28.21 ± 2.082	–5.247	34	<0.001
MoCA (mean ± SD)	13.78 ± 4.045	28.07 ± 1.592	–12.629	34	<0.001

ST means patients with stroke, and HI means healthy individual. MMSE presents the mini-mental state examination; MoCA presents the Montreal cognitive assessment.

Informed consent was signed by the patient himself or by his immediate family. The study was approved by the Ethics Committee of Yuebei People’s Hospital Affiliated with Shantou University Medical College (Ethical approval number: SUMC-IRB-2020) on 29 April 2020 and registered in the Chinese Clinical Trials Registry (registration No. ChiCTR2000032804).

### Research process

**FIGURE 1:**
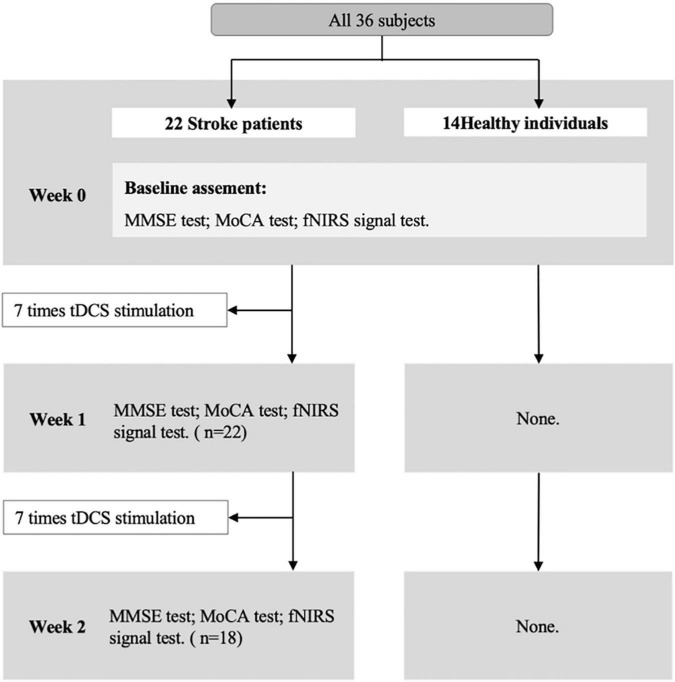
Research process.

The Montreal Cognitive Assessment (MoCA) and the Mini-Mental State Examination (MMSE) were used to detect cognitive states before and after tDCS. The fNIRS with VFT was performed before and after tDCS. The tDCS intervention was taken only in the stroke group. The research process is shown in Figure 1.

### Transcranial direct current stimulation

Treatment procedure: Patients with stroke receive tDCS treatment 7 times a week for 2 weeks (once a day). The duration of each tDCS application is 30 min. In the tDCS setting, the anode was attached over the F3 (10–20 EEG electrodes) (48) with 2 mA intensity. The return electrode was positioned over the F4 (10–20 EEG electrodes). After each tDCS application, the patients completed a brief adverse effect questionnaire (49). Before the tDCS, after the 7 times tDCS, and after the 14 times tDCS, the participants were tested for cognitive and cerebral hemodynamic signals with fNIRS, respectively.

### Cognitive function measurement

All subjects were treated with comprehensive clinical evaluation. The MMSE is the most often used short screening tool for providing a clear impression of overall cognitive decline and checking the recovery of cognitive function after stroke (50, 51). Its validity has been demonstrated in patients with stroke (52). The MoCA can be used to assess people’s cognitive ability, including aspects of memory, executive function, attention, concentration, language, abstract reasoning, and orientation, with a maximum score of 30 (53, 54). Its validity has been demonstrated in patients with stroke (54).

### Functional near-infrared spectroscopy measurement

#### Verbal fluency task process

**FIGURE 2:**
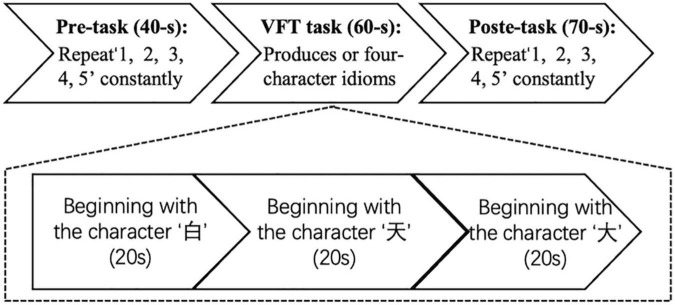
Experimental flowchart. The experiment has three procedures: 40-s pre-task, 60-s verbal fluency task (VFT) task, and 70-s post-task.

The VFT flow is shown in Figure 2. The task time was about 170 s. The first 40 s and the last 70 s of the experiment are used to collect the resting-state signal of the participants. During these two stages, the participants are required to count from one to five repeatedly. During the second stage, the participants performed VFT of three consecutive word-generating tasks, each of which lasted 20 s.

#### Functional near-infrared spectroscopy signal acquisition

**FIGURE 3:**
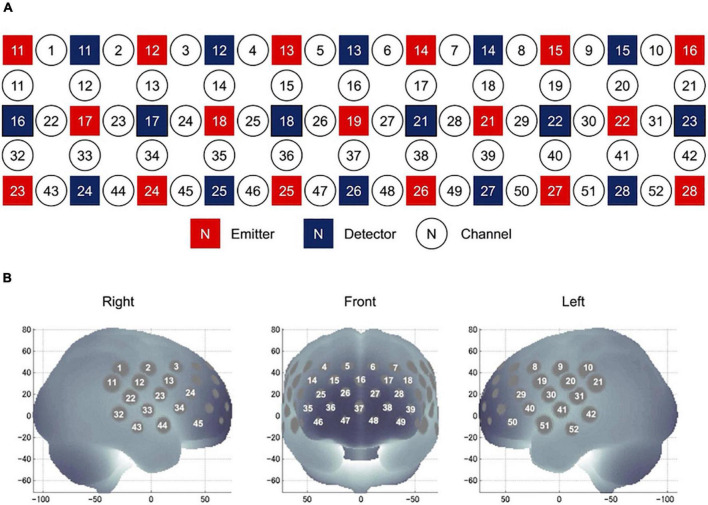
Location of NIRS channels. **(A)** Arrangement of the 17 emitters and 16 detectors and definition of the channels. **(B)** The anatomical site corresponding to each channel.

During the VFT experiment, we used a 52-channel fNIRS device (ETG-4000 Optical Topography System) to estimate changes in regional cortical Hb concentration during the cognitive activation task, as described previously (55). The probes (17 emitters and 16 detectors, alternating) were fixed using 3 × 11 thermoplastic shells with an inter-optode distance of 3.0 cm. Each adjoining pair of an emitter and detector was referred to as a “channel,” resulting in 52 channels in total (Figure 3A). The lowermost probes were positioned along the Fp1–Fp2 line according to the International 10–20 EEG system (Figure 3B). The probes can measure Hb values bilaterally from the prefrontal and temporal surface regions at a depth of 20–30 mm from the scalp. This depth range corresponds roughly to the surface of the cerebral cortex. NIRS measures relative changes in oxy- and deoxy-Hb concentrations (in mM) using two wavelengths (695 and 830 nm) of near-infrared light based on the modified Beer–Lambert law (56). The sampling frequency is 10 Hz. The experiment was carried out in a quiet room with an appropriate temperature. After the experiment, the subjects were asked to sit quietly in the most comfortable position and minimize eye and other body movements.

Nine ROIs were set at the following regions (57): right superior frontal cortex (rSFC) (detected by the 1st, 2nd, 11th, and 12th channels), right superior temporal cortex (rSTC) (detected by the 22nd, 23rd, 32nd, 33rd, 43rd, and 44th channels), right dorsolateral prefrontal cortex (rDLPFC) (detected by the 3rd, 4th, 13th, 14th, 15th, 24th, and 25th channels), right ventrolateral prefrontal cortex (rVLPFC) (detected by the 34th, 35th, 45th, and 46th channels), medial prefrontal cortex (mPFC) (detected by the 5th, 6th, 16th, 26th, 27th, 36th, 37th, 38th, 47th, and 48th channels), left dorsolateral prefrontal cortex (lDLPFC) (detected by the 7th, 8th, 17th, 18th, 19th, 28th, and 29th channels), left ventrolateral prefrontal cortex (lVLPFC) (detected by the 39th, 40th, 49th, and 50th channels), left superior frontal cortex (lSFC) (detected by the 9th, 10th, 20th, and 21st channels), and left superior temporal cortex (lSTC) (detected by the 30th, 31st, 41st, 42nd, 51st, and 52nd channels).

### Data processing of functional near-infrared spectroscopy data

The fNIRS data were analyzed using the Homer2 package in MATLAB and customized MATLAB-based script (58). First, the raw light intensity file has been converted to homer2 file format (.nirs). Then, the raw fNIRS data were first converted to optical density (function: *hmrIntensity2OD*), and using the manufacturer’s recommendations, channels with a variation coefficient greater than 15% are considered bad channels and deleted from the analysis. A wavelet transform was used to correct motion artifact (function: *hmrMotionCorrectWavelet*) using the default interquartile range (0.1), as this is optimal for motion correction. Any remaining motion artifact was then removed through the motion artifact detection tool (function: *hmrMotionArtifact*, tMotion = 0.5, tMask = 1.0, STDEVthresh = 20, AMPthresh = 5.0). The signal was then bandpass-filtered (function: *hmrBandpassFilt*, hpf = 0.000, lpf = 0.10) to remove baseline drift and physiological noise. Finally, the concentration changes of oxy-Hb were then computed according to the modified Beer–Lambert law. Additionally, we took the final 5 s of the pre-task rest period as the baseline. The oxy-Hb values were then saved as text files for each subject. Finally, the oxy-Hb time series for each subject was z-scored by channel. Note that post-analysis in this study was solely based on oxy-Hb, since the oxy-Hb signal is known to be more robust and sensitive than deoxy-Hb to task-associated changes (59–61).

Based on the MATLAB-based Nirs_Kit software (62), we used the GLM model (63, 64), using mission oxygenated hemoglobin (oxy-Hb) β value minus rest oxygenated hemoglobin β value, representing the cortical activation. Then, we used an independent sample t-test to compare the cortical activation between patients with stroke and healthy control groups and used the ANOVA test to compare the cortical activation among before tDCS, after 7 times tDCS, and after 14 times tDCS in the stroke group.

### Data analysis of functional connectivity

As stroke can disrupt FC and cause brain-wide network changes (65), it is important to investigate brain-wide network dynamics during post-stroke recovery. This study uses a technology similar to the existing research to study FC (66, 67). The Nirs_Kit toolbox was used to conduct statistical comparisons (62). The primary threshold (test statistic) for electrode pairs was set to a conservative value of *t* = 3.1 (equivalent to *p* = 0.001) to ensure that only highly robust and reliable connectivity differences would be compared at the cluster level (68). A value of *p* < 0.05 (two-tailed) was used as the secondary significance threshold for family-wise corrected cluster analysis (5,000 permutations) (68). Subsequent visualization of brain networks was performed using the BrainNet viewer toolbox (69).

## Results

### Mini-mental state examination and Montreal cognitive assessment

First, based on previous studies and clinical observations, we assume that the cognitive function of the stroke group is lower than that of the healthy control group. We used the independent sample t-test to compare the MMSE and MoCA of the stroke group and the healthy control group. The results show that both MMSE score (*m* = 20 ± 4.739) and MoCA score (*m* = 13.78 ± 4.045) in the stroke group were lower than that in the healthy control group (*m* = 28.21 ± 2.082), (*m* = 28.07 ± 1.592), [*t*_(34)_ = –5.247, *p* < 0.001], [*t*_(34)_ = –12.629, *p* < 0.001]. This shows that compared with the healthy control group, the cognitive function of the stroke group has decreased.

**FIGURE 4:**
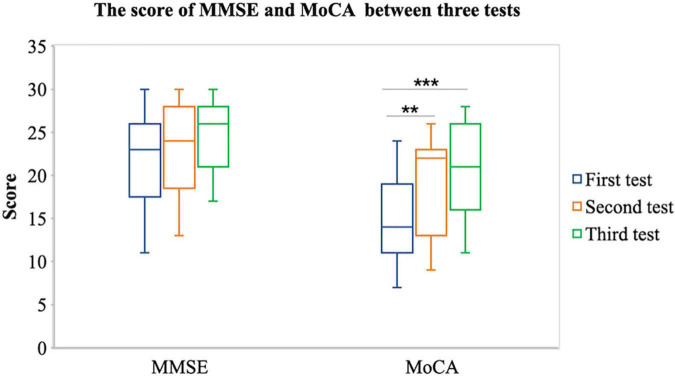
Comparison of MMSE and MoCA between the first, second, and third tests. ^**^P < 0.05 and ^***^P < 0.001.

Second, we assume that after the tDCS, the cognitive function of patient with stroke will increase. Therefore, we conducted an ANOVA test on MMSE and MoCA scores of three tests in the stroke group. The results showed that there was a significant difference in MoCA score among before tDCS, after 7 times tDCS, and after 14 times tDCS [*F*_(1,58)_ = 11.860, *p* < 0.001]; the results of multiple comparisons show that the MoCA score after 7 times tDCS (*m* = 18.10 ± 5.612) is significantly higher than that before tDCS (*m* = 14.38 ± 4.631) [*p* = 0.025]; the results of multiple comparisons show that the MoCA score after 14 times tDCS (*m* = 20.17 ± 5.426) is significantly higher than that before tDCS (*m* = 14.38 ± 4.631) [*p* < 0.001], as shown in Figure 4. There were marginally significant differences in MMSE score among before tDCS, after 7 times tDCS, and after 14 times tDCS [*F*_(1,58)_ = 3.782, *p* = 0.057]; the results of multiple comparisons show that the MMSE score after 14 times tDCS (*m* = 24.44 ± 4.105) was marginally significantly higher than that before tDCS (*m* = 21.33 ± 5.453) [*p* = 0.057]. This shows that after 7 times tDCS and 14 times tDCS, the cognitive function measured by MoCA of patients with stroke has been significantly improved.

### Cortical activation

**FIGURE 5:**
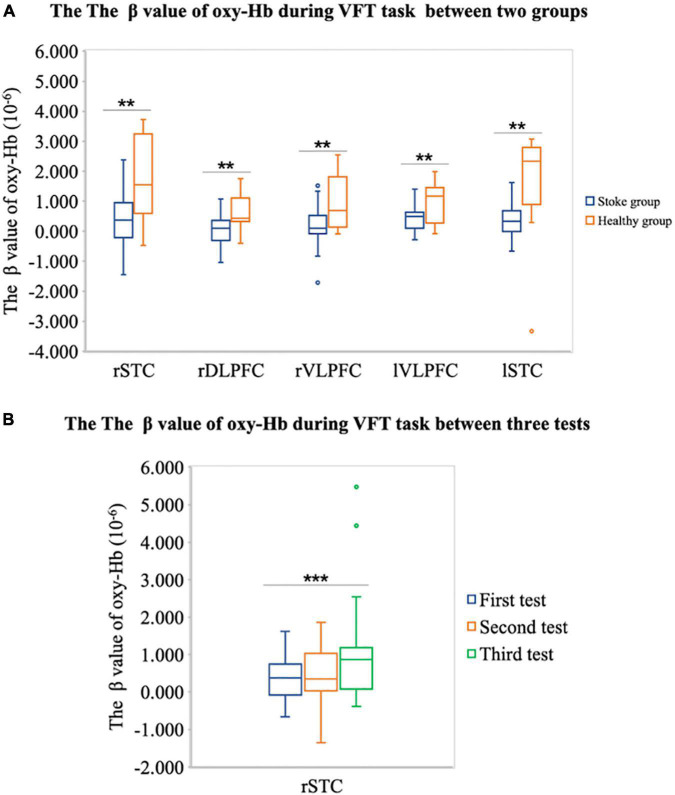
**(A)** Difference of the β value of oxy-Hb during VFT between the stroke group and the healthy individual group on rSTC, rDLPFC, rVLPFC, and lVLPFC areas. **(B)** The difference of the β value of oxy-Hb on the right superior temporal cortex (rSTC) during VFT between three tests in patient with stroke. ^**^P < 0.05 and ^***^P < 0.001.

First, based on the phenomenon that the cognitive function of the stroke group is lower than that of the healthy control group, we assume that the cortical activation of the stroke group is lower than that of the healthy control group. Therefore, we compared the cortical activation of the stroke group and the healthy control group in nine brain cortexes, including rSFC, rSTC, rDLPFC, rVLPFC, mPFC, lDLPFC, lVLPFC, lSFC, and lSTC. The independent sample *t*-test results show that the β value of the stroke group was lower than that of the healthy control group in the rSTC [*m_(*stroke*)_* = 0.510 ± 0.970, *m_(control)_* = 1.705 ± 1.381, *t*_(34)_ = –2.966, *p* = 0.006], rDLPFC [*m_(*stroke*)_* = 0.035 ± 0.522, *m_(control)_* = 0.592 ± 0.599, *t*_(34)_ = –2.852, *p* = 0.008], rVLPFC [*m_(*stroke*)_* = 0.163 ± 0.711, *m_(control)_* = 0.906 ± 0.894, *t*_(34)_ = –2.683, *p* = 0.011], lVLPFC [*m_(*stroke*)_* = 0.400 ± 0.391, *m_(control)_* = 0.951 ± 0.674, *t*_(34)_ = –2.677, *p* = 0.016], and lSTC [*m_(*stroke*)_* = 0.360 ± 0.635, *m_(control)_* = 1.641 ± 1.754, *t*_(34)_ = –2.533, *p* = 0.023], as shown in Figure 5A. This shows that compared with the healthy control group, the brain cortical activation of the stroke group during the VFT has decreased in a wide range of brain regions.

**FIGURE 6:**
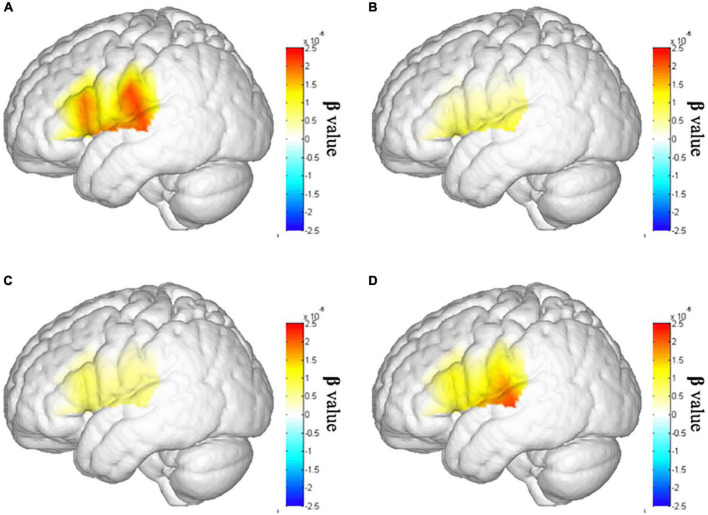
Brain activation map of the left superior temporal cortex (lSTC). **(A)** Brain activation map of lSTC in the healthy individual group. **(B)** Brain activation map of lSTC in the stroke group before tDCS. **(C)** Brain activation map of lSTC in the stroke group after seven times tDCS. **(D)** Brain activation map of lSTC in the stroke group after 14 times tDCS.

Second, we assume that after the tDCS, the cortical activation in lSTC of patient with stroke will increase. Therefore, we conducted an ANOVA test on cortical activation in lSTC of three tests in the stroke group. The results showed that there were significant differences in the lSTC in the stroke group among before tDCS, after seven times tDCS, and after 14 times tDCS [*F*_(1,58)_ = 4.488, *p* = 0.038], as shown in Figure 6; the results of multiple comparisons showed that the β value on the lSTC during VFT after 14 times tDCS (*m* = 0.360 ± 0.643) was significantly higher than that before tDCS (*m* = 1.122 ± 1.576) [*p* = 0.038], as shown in Figures 5B, 6. This shows that after 14 times tDCS, the cortical activation in the lSTC in the stroke group has been significantly improved.

Third, we further explored whether the cortical activation of the patient with stroke has recovered to normal level after 14 times tDCS. we assume that there is no difference between the cortical activation of patients with stroke after 14 times tDCS and that of the healthy control group. The independent sample t-test results show that there is no difference between the cortical activation on all cortexes in patients with stroke after 14 times tDCS and that in the healthy control group. This shows that after 14 times tDCS, the cortical activation of patients with stroke has recovered to the same level as that of healthy peers.

### Functional connectivity

**FIGURE 7:**
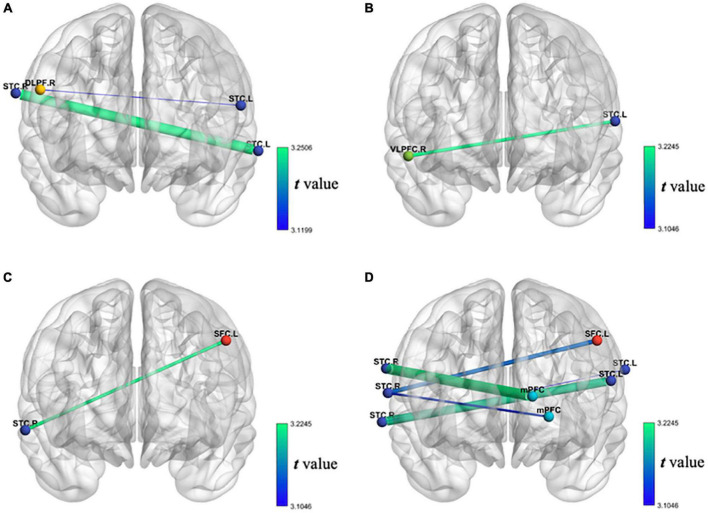
**(A)** Decrease of FC of oxy-Hb during VFT in patients with stroke compared with the healthy individual group; **(B)** the decrease of FC of oxy-Hb during VFT of the first test compared with the second test in patients with stroke; **(C)** the increase of FC of oxy-Hb during VFT of the first test compared with the third test in patients with stroke; **(D)** the increase of FC of oxy-Hb during VFT of the second test compared with the third test in patients with stroke.

First, as the balance between the cerebral hemispheres of patients with stroke is destroyed (6), we propose the assumption that the FC between bilateral cerebral hemispheres in the stroke group is lower than that in the healthy control group. We used the correlation between bilateral cerebral hemispheric channels’ oxy-Hb during VFT to represent FC. The result shows that the FC between rSTC and lSTC in the stroke group is significantly lower than that in the healthy control group. The FC between rDLPFC and lSTC in the stroke group is significantly lower than that in the healthy control group, as shown in Figure 7A. This shows that compared with the healthy control group, the FC between bilateral cerebral hemispheres of the stroke group during the VFT has decreased, especially the FC between the lSTC and the rSTC and the FC between the lSTC and the rDLPFC.

Second, we assume that after the tDCS, the FC between bilateral cerebral hemispheres of patient with stroke will increase. Similarly, we used the correlation between bilateral cerebral hemispheric channels’ oxy-Hb during VFT to represent FC. The unexpected results showed that the FC between rVLPFC and lSTC after seven times tDCS is significantly lower than that before tDCS in the stroke group, as shown in Figure 7B, but the expected results showed that the FC between lSFC and rSTC after 14 times tDCS is significantly higher than that before tDCS in the stroke group, as shown in Figure 7C. Similarly, the expected results showed that the FC between lSFC and rSTC after 14 times tDCS is significantly higher than that after seven times tDCS in the stroke group. The FC between the mPFC and the rSTC after 14 times tDCS is significantly higher than that after seven times tDCS in the stroke group. The FC between the lSTC and rSTC after 14 times tDCS is significantly higher than that after seven times tDCS in the stroke group, as shown in Figure 7D. This shows that after 14 times tDCS, the FC between bilateral cerebral hemispheres of the stroke group during the VFT has increased. In addition, during the whole 14 times of tDCS, the stimulation from the 7th to the 14th time was the most effective.

## Discussion

Post-stroke cognitive impairment is a common complication after stroke (1, 2). After a stroke, people have been found to have decreased brain cortical activation (4, 5) and FC (10–12), which is associated with persistent functional impairment. Therefore, regulating neural activity in stroke patients with cognitive impairment is critical. tDCS is a promising tool for increasing cortical activity (20) and FC (29–31). fNIRS is a non-invasive optical technique that can indirectly observe the activation of the cerebral cortex (33). Therefore, we investigated the effect of tDCS with anodes and cathodes on F3 and F4 electrodes on the rehabilitation of cognitive impairment in patients with stroke using fNIRS technology. This study’s results showed that the cognitive function of patients with stroke was lower than that of healthy individuals but improved after tDCS. The cortical activation of patients with stroke was lower than that of healthy individuals in the lSTC, rSTC, rDLPFC, rVLPFC, and lVLPFC cortical regions; cortical activation increased in the lSTC cortex after tDCS. The FC between the cerebral hemispheres of patients with stroke was lower than that of healthy individuals and increased after 14 tDCS sessions. This shows that the cognitive and brain function of patients with mild-to-moderate stroke were damaged but could recover after tDCS.

Cognitive function in older adult patients with stroke is impaired and can be improved after tDCS. This research shows that the cognitive function identified by the MMSE and the MoCA scores in patients with stroke was lower than in healthy peers but improved after tDCS. Post-stroke cognitive impairment is a known complication after stroke and a critical risk predictor of a decline in quality of life (1–3). Some researchers applied tDCS to stimulate the brain cortex. They found that cognitive functions, such as naming accuracy (18), the scores of attention and concentration, figural memory, logical reasoning, reaction behavior (2), working memory (20, 70), and executive function (27), improved in patients with stroke. Therefore, it can be inferred that the tDCS intervention is an effective treatment to improve cognitive function for stroke patients with cognitive impairment.

Cortical brain activation in older adult patients with stroke is decreased and can be re-activated after tDCS. This research shows that cortical activation in patients with stroke was lower than in healthy individuals in the cortices of the lSTC, rSFC, rDLPFC, rVLPFC, and lVLPFC. This increased to the same level as normal peers in the lSTC after tDCS. Consistent with the results of this study, previous studies have also found that the decreased extent of cortical activation in patients with stroke is related to the degree of functional damage (4, 5). Post-stroke, the lesions may be widespread in the frontal and temporal regions (45, 46). Fortunately, a series of studies have found that tDCS can alter the excitability of the cortex (21, 24–28, 71). Anodic stimulation increases cortical excitability, whereas cathodic stimulation decreases it (71). In addition, regarding the IHI state after stroke, tDCS can increase ipsilateral excitability and reduce contralateral excitability due to the destruction of IHI after stroke (24). This study placed the anodes and cathodes on F3 and F4 electrodes (10–20 EEG electrodes) with 2 mA intensity and found that the cortical activation of patients with stroke increased in the lSTC cortex after tDCS. Therefore, tDCS positively affects the recovery of cerebral cortical function in patients with stroke (24, 28).

The brain FC between the bilateral cerebral hemispheres of older adult patients with stroke is decreased but can be re-connected after tDCS. This research showed that the FC between the bilateral cerebral hemispheres in patients with stroke was lower than in healthy individuals and increased after 14 tDCS sessions. Consistent with the results of this study, previous studies found that the damage to neural systems caused brain-wide network changes. The balance between the cerebral hemispheres of patients with stroke is destroyed (6) when a stroke occurs, leading to the disinhibition of the contralateral cortex and excessive activation, further reducing brain function (7–9). The promising findings are that tDCS can widely increase this interhemispheric connectivity in patients with stroke (29–31).

This research makes an important contribution to the diagnosis and effective treatment of brain function in patients with cognitive impairment after stroke. Theoretically, this research confirms that patients with stroke have brain function damage (4, 5, 45, 46). Second, this study expanded the research range of brain function damage to the connectivity between the bilateral cerebral hemispheres. Based on the theoretical model of competition inhibition between the bilateral cerebral hemispheres (6–9), this study found that in patients with stroke, the FC between the bilateral cerebral hemispheres is abnormally reduced. More importantly, based on previous studies, this study confirmed that tDCS is an effective intervention for post-stroke cognitive impairment. In placing the anodes and cathodes on F3 and F4 electrodes during tDCS, we can effectively improve cognitive impairment after stroke, re-activate the cerebral cortex, and re-connect the FC of the bilateral cerebral hemispheres. In this study, we found that increased cortical activation and FC between bilateral cerebral hemispheres measured by fNIRS are promising biomarkers to assess the effectiveness of tDCS in patients with stroke. These two neural indicators can be used clinically to measure cerebral function recovery in stroke patients with cognitive impairment.

## Limitations

This study had some limitations. There was no control group with healthy older adult peers in the tDCS intervention study, and the use of drugs in treating patients with stroke has not been controlled. Future researchers can verify these results using more cognitive task paradigms. Moreover, they can explore the mechanism of action of tDCS and drug use in treating patients with stroke.

## Conclusion

In conclusion, post-stroke cognitive impairment is a common complication of stroke. After a stroke, people have been found to have abnormal cortical activation in the brain, which is associated with persistent cognitive impairment. tDCS is a promising tool for assessing changes in cortical activation. fNIRS is a non-invasive optical technique that can indirectly observe cortical activation. This study investigated the effect of tDCS on the rehabilitation of cognitive impairment in patients with stroke using fNIRS technology. This study’s results showed that the cognitive ability of patients with stroke, measured on the MMSE and MoCA, was lower than that of healthy individuals but was improved after tDCS. The cortical activation of patients with stroke was lower than that of healthy individuals on the lSTC, rSTC, rDLPFC, rVLPFC, and lVLPFC cortical regions; this increased in the lSTC cortex after tDCS. The FC between the cerebral hemispheres of patients with stroke was lower than that of healthy individuals and increased after 14 tDCS. This shows that the cognitive and brain function of mild-to-moderate patients with stroke were damaged but could be recovered after tDCS. Increased cortical activation and increased FC between the bilateral cerebral hemispheres measured by fNIRS are promising biomarkers to assess the effectiveness of tDCS in stroke.

## Data availability statement

The raw data supporting the conclusions of this article will be made available by the authors, without undue reservation.

## Ethics statement

The studies involving human participants were reviewed and approved by Ethics Committee of Yue Bei People’s Hospital. The patients/participants provided their written informed consent to participate in this study.

## Author contributions

PW, YZ, CY, KH, and HL participated in this study and proposed an experimental design. PW, CY, TZ, and KH participated in the experimental process of this study. TZ and CY participated in the data analysis of this study. TZ, MX, and CY participated in the writing of this study. PW, YZ, CY, and TZ participated in the modification of this study. All authors contributed to the article and approved the submitted version.
